# A High-Speed Vision-Based Sensor for Dynamic Vibration Analysis Using Fast Motion Extraction Algorithms

**DOI:** 10.3390/s16040572

**Published:** 2016-04-22

**Authors:** Dashan Zhang, Jie Guo, Xiujun Lei, Changan Zhu

**Affiliations:** Department of Precision Machinery and Precision Instrumentation, University of Science and Technology of China, Hefei 230027, China; zhangds@mail.ustc.edu.cn (D.Z.); guojiegj@mail.ustc.edu.cn (J.G.); leijun@mail.ustc.edu.cn (X.L.)

**Keywords:** vision-based measurement, high-speed camera system, image registration, phase correlation, subpixel accuracy improvement

## Abstract

The development of image sensor and optics enables the application of vision-based techniques to the non-contact dynamic vibration analysis of large-scale structures. As an emerging technology, a vision-based approach allows for remote measuring and does not bring any additional mass to the measuring object compared with traditional contact measurements. In this study, a high-speed vision-based sensor system is developed to extract structure vibration signals in real time. A fast motion extraction algorithm is required for this system because the maximum sampling frequency of the charge-coupled device (CCD) sensor can reach up to 1000 Hz. Two efficient subpixel level motion extraction algorithms, namely the modified Taylor approximation refinement algorithm and the localization refinement algorithm, are integrated into the proposed vision sensor. Quantitative analysis shows that both of the two modified algorithms are at least five times faster than conventional upsampled cross-correlation approaches and achieve satisfactory error performance. The practicability of the developed sensor is evaluated by an experiment in a laboratory environment and a field test. Experimental results indicate that the developed high-speed vision-based sensor system can extract accurate dynamic structure vibration signals by tracking either artificial targets or natural features.

## 1. Introduction

Engineering structures including bridges and buildings are inevitably exposed to various external loads, such as traffic, earthquakes and gusts during their lifetime. These external loads may induce structure damage and lead to life-threatening materials’ failure. Conventional sensors like accelerometers [1] are widely employed in monitoring system to obtain valuable vibration information for mechanical analysis or structure safety evaluation. However, conventional sensors can only obtain vibration acceleration signal, which does not provide an intuitionistic exhibition of the actual vibration. Besides that, the installation of contact sensors is sometimes difficult and the additional mass of sensors may change the behavior of structures in some precise measurements. Non-contact measurement techniques, such as speckle photography [2], hologram interferometry [3] and laser Doppler vibrometer [4], have been developed for years as effective alternatives to traditional measurement devices. However, most of the non-contact equipment require high costs and are composed of complex structures; thus, these systems can hardly be used widely in practical applications.

With the development of image sensor and optics lens, the vision-based approaches have becoming burgeoning alternatives to this non-contact measurement equipment for their relatively low cost and flexibilities in structures [5,6]. Benefiting from the development of image sensors and high-performance zoom lenses, vision-based measurement equipment has been widely used in many research fields and industrial areas such as vibration analysis [7,8], condition monitoring [9,10,11,12], human motion analysis [13,14] and underwater measurement [15]. Meanwhile, the vision-based techniques were successfully used for displacement measurement of various engineering structures and obtain satisfactory results [16,17,18,19,20]. Quan *et al.* [21] achieved three-dimensional displacement measurement by using the two-dimensional digital image correlation (DIC) technique. Kim *et al.* [22] proposed a vision-based monitoring system based on DIC to evaluate the cable tensile force of a cable-stayed bridge. Lee *et al.* [23] developed a vision-based dynamic rotational angle measurement system for large civil structures using image processing techniques with pre-measured calibration parameters. Park *et al.* [24] realized the displacement measurement for high-rise building structures using partitioning approach. Fukuda *et al.* [25] proposed a camera-based sensor system with which a robust object search algorithm was employed to measure the dynamic displacements of large-scale structures. Traditional vision-based sensor systems usually compose of commercial digital cameras and video processing modules. However, ordinary digital cameras can hardly meet the requirement of high frequency vibration analysis because of the limitation of low sampling frame rate. To overcome the restriction, high-speed camera systems [26,27,28] with 1000 fps or even higher frame fate have been developed and applied in recent years. Chen *et al.* [27] realized modal identification of simple structures by applying phase-based optical flow and motion magnification techniques on high-speed videos. Cha *et al.* [28] proposed a damage detection methodology based on phase-based motion extraction and unscented Kalman filter and used this method to detect damages in structural system using a high-speed vision system.Through these methods [27,28], the operational deflection shapes of structure can be identified and visualized.

High-speed cameras have strict efficiency demands for image processing techniques to realize real-time measurement. Phase-based optical flow [27,28,29,30] provides an efficient approach to estimate velocity component at all points in the image sequences. However, spatial filtering of phase-based optical flow is time-consuming, and the determination of coefficients for spatial band-pass filter is complex. These characteristics make phase-based optical flow a more appropriate choice for post-processing. Conventional motion extraction algorithms based on image feature matching are commonly subjected to many shortcomings, such as complex process and multi-parameter adjustment. Most template matching algorithms, *i.e.*, sum of squared differences (SSD) or normalized cross-correlation [31] (NCC), need to scan a template on the whole image to find the best matching. Moreover, since the minimal unit in a video image is one pixel, these algorithms can only achieve integer level resolution. Such performances in accuracy and efficiency are far from satisfaction in many practical applications, particularly for long-distance or small-amplitude measurements. In the research field of image registration, different subpixel level algorithms in both time and frequency domain have been proposed, such as the enhanced correlation coefficient (ECC) algorithm [32] and the upsampled cross-correlation (UCC) algorithm [33]. However, either the image interpolation [34,35] or fast Fourier transform (FFT) upsampling operation [36] will increase heavy computation burden, which makes them not very suitable for real-time measurement in high-speed sensor system.

Two efficient subpixel level motion extraction algorithms are proposed in our research with consideration for both efficiency and accuracy. Based on the normalized root-mean-square error [36] (NRMSE) theory, rough motion estimation is able to be accomplished by using phase correlation technique. The integer level motion extraction results are extracted by locating maximum values in cross-correlation matrices between the two-dimensional discrete Fourier transform (2D DFT) of the base template and the object images. Although the phase correlation algorithm is theoretically more complicated and time-consuming than the time-domain cross-correlation algorithm, the integer level motion extraction can be achieved in only one calculation. Two efficient subpixel refinement approaches, namely the modified Taylor approximation refinement and the localization refinement, are applied to improve the rough search results to subpixel level. Without intensity interpolation or cross-correlation upsampling, these two proposed algorithms can execute with high degree of automation and run much faster than the compared algorithms. Quantitive analysis of the proposed algorithms is given through a simulation test. Two experiments under laboratory and realistic conditions are carried out to evaluate the performance of developed sensor system.

The rest of the paper is organized as follows: Section 2 introduces the components and capability parameters of the high-speed vision-based sensor system. Section 3 presents theories of phase correlation technique and two subpixel refinement approaches by using a simulation test and gives the quantitive analysis. Section 4 evaluates the performance of a developed sensor system through a motion platform experiment and a field test. Discussions and outlooks are also presented in this section. Section 5 concludes the whole article.

## 2. High-Speed Visual Measuring System

**Figure 1:**
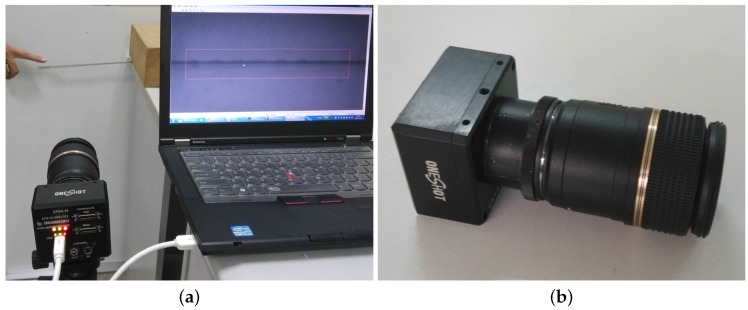
High-speed visual measuring system. (**a**) experimental setup; (**b**) camera head and optical lens.

The developed high-speed vision-based sensor system is mainly composed of a camera head, a zoom lens and a notebook computer (Intel Core processor, Santa Clara, CA, USA; 2.9 GHz; 2.75 GB RAM) as shown in Figure 1. A high-performance charge-coupled device (CCD) which can capture 8-bit gray-scale images (300 pixel × 300 pixel) with, at most, 1000 fps is integrated into the camera head as the image receiver. The telescopic lens with large zooming capability can meet the measurement requirement at different distances. A USB 3.0 interface is applied to ensure stable data transfer between the camera head and the notebook computer. The algorithms proposed in this paper have been integrated into the developed sensor system based on Qt and open source computer vision (OpenCV) libraries. The programmed motion extraction software consists of camera control module, calibration module and video analysis module. The control module is responsible for adjusting parameters of the camera head such as contrast, brightness and exposure time. The calibration module is used to calculate the actual size that single pixel occupied on the target. Image sequence captured by the camera head will be transferred to the analysis module for real-time analysis.

In the measurement procedure, a pre-installed target panel on distant objects will be very helpful to the motion extraction accuracy. If the target panel is unavailable because of the limitation of the measurement environment, the distinct surface features, such as textures or edges for which actual dimensions are known, can also be used for camera calibration.

## 3. Methodology

**Figure 2:**
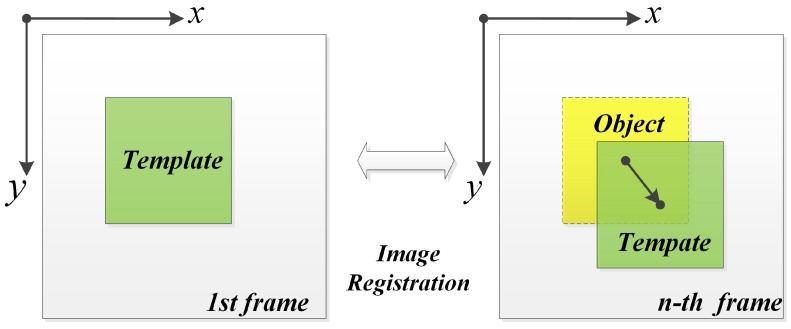
Basic procedure of the implementation.

In this study, two efficient methods are proposed and applied in the developed visual measuring system to refine integer level motion extraction result to subpixel level. The basic procedure of the algorithms is shown in Figure 2. An initial area to be tracked is defined as a template in the first image of a sequence of the captured video frames. Object images are obtained by cutting the successive images at the same position of the template. The motion extraction between template image and every object image can be realized by using improved image registration algorithms. In these algorithms, the integer level motion extraction is realized by using phase correlation technique on the integer grid at first. Two efficient refinement approaches are proposed to refine the integer level motion extraction result on the subpixel level. Quantitive analysis and comparisons are given at the end of this section.

### 3.1. Normalized Root-Mean-Square Error

Motion extraction will be conducted by using a phase correlation technique on the integer grid at first. Given a coordinate translation $\left(x_{0},y_{0}\right)$ and a multiplicative constant factors *α*, the normalized root-mean-square error (NRMSE) between the template image f(x,y) and object image g(x,y) can be defined as

(1) $$E^{2}=\min_{\alpha ,x_{0},y_{0}}=\frac{\sum_{x,y}{\left|\alpha g\left(x-x_{0},y-y_{0}\right)-f\left(x,y\right)\right|}^{2}}{\sum_{x,y}{\left|f(x,y)\right|}^{2}}$$

where summations are taken over all image points (x,y). For a given translation $\left(x_{0},y_{0}\right)$, the constant factor *α* can be solved as

(2) $$\alpha =\frac{r_{fg}\left(x_{0},y_{0}\right)}{\sum_{x,y}{\left|g(x,y)\right|}^{2}}$$

where

(3) $$r_{fg}\left(x_{0},y_{0}\right)=\sum_{x,y}f\left(x,y\right)g^{*}\left(x-x_{0},y-y_{0}\right)=\sum_{x,y}F\left(u,v\right)G^{*}\left(u,v\right)exp\left[i2\pi \left(\frac{ux_{0}}{M}+\frac{uy_{0}}{N}\right)\right]$$

in which $r_{fg}\left(x_{0},y_{0}\right)$ is the cross-correlation of f(x,y) and g(x,y); the asterisk donates complex conjugation; *N* and *M* are the template dimensions; F(u,v); and T(u,v) represent the two-dimensional discrete Fourier transform (2D DFT) of their lowercase counterparts, for example,

(4) $$F\left(u,v\right)=\sum_{x,y}\frac{f(x,y)}{\sqrt{MN}}exp\left[-i2\pi \left(\frac{ux_{0}}{M}+\frac{uy_{0}}{N}\right)\right]$$

With the derivation above, Equation (1) can be simplified by inserting the constant factor into the NRMSE metric:

(5) $$\min_{\alpha }E^{2}=1-\frac{\max_{x_{0},y_{0}}{\left|r_{fg}\left(x_{0},y_{0}\right)\right|}^{2}}{\sum_{x,y}{\left|f(x,y)\right|}^{2}\sum_{x,y}.{\left|g(x,y)\right|}^{2}}$$

Then, the NRMSE minimization problem is transformed into locating the maximum value in the cross-correlation matrix $r_{fg}\left(x_{0},y_{0}\right)$ in the frequency domain.

Since the location information of the maximum cross-correlation value has a significant relationship with the coordinate translation, this image registration algorithm provides a simple and intuitive way to find the movement between two images. For a vibration video containing a series of images, the motion information can be easily extracted when the location of maximum cross-correlation value is searched repeatedly between the 2D DFT of the base image and every object image in the following video sequence. According to the analysis above, the procedure of maximum cross-correlation (MCC) motion extraction algorithm in the 2D frequency domain is summarized as follows:

1. Capture the video and make sure the moving target is always in the photographic range.
2. Cut the template image in the first frame and cut the object images to be aligned in the following video image sequence at the same position.
3. Calculate the cross-correlation matrices between the 2D DFT of the template image and every object image. Then, record the coordinates of the maximum value in every cross-correlation matrix.

Eventually, the image motion in the horizontal and vertical directions can be calculated using these coordinate information. For the reason that the change of coordinate in obtained cross-correlation matrices represent pixel movement in practice, the MCC-based motion extraction algorithm provides an effective approach to realize the displacement measurement. A simulation test is provided here to demonstrate the effectiveness of the MCC-based motion extraction procedure using Matlab R2015a. The simulation gives a simple vignetting black circle, which has a diameter of 240 pixels. This black circle is programmed to move along the following ellipse equation:

(6) x(t)=10cos(2πft),y(t)=6cos(2πft)

**Figure 3:**
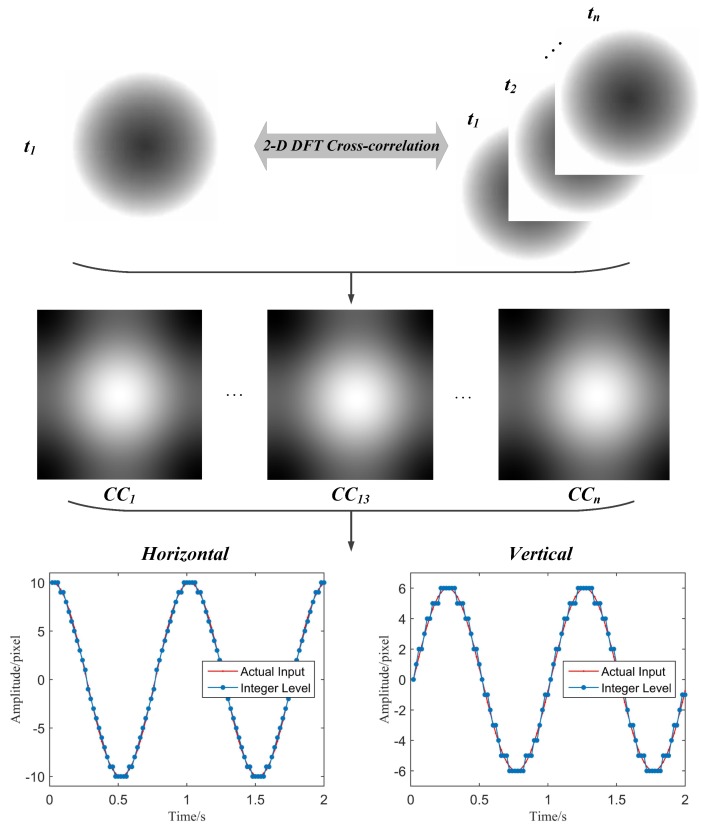
Procedure of maximum cross-correlation (MCC) motion extraction algorithm in 2D frequency domain.

The rotation frequency *f* of the virtual circle is set to be 1 Hz and the sampling frequency $f_{s}$ is 50 Hz. The MCC-based motion extraction algorithm is applied to capture the vibration signals of the black circle in both horizontal and vertical directions. The implementation procedure of the MCC-based algorithm and the motion extraction results is illustrated in Figure 3. The template image and object images can be selected in the first frame and successive frames. Then the cross-correlation matrices (CC) between 2D DFT of the template image and every object image are calculated. Finally, the integer level motion information can be obtained by locating the maximum value in each CC matrix. The results indicate that the MCC-based algorithm successfully extract the motion signals of the black circle in the simulation. The wave shapes in both horizontal and vertical directions match well with the programmed actual input.

However, since the coordinates of the maximum cross-correlation values are always integers, the MCC-based motion extraction only can return pixel-level displacement results between the template image and the object images. This deficiency certainly will affect the measurement accuracy and lead to step-type motion extraction results. It is particularly to point out that the step-type influence is especially significant in long-distance or small-amplitude measurement. Because of the resolution limitation of CCD, the useable pixels per inch (PPI) decreases in both long-distance and small-amplitude measuring conditions. Measuring error less than one pixel may seriously influence the extraction accuracy of vibration signal. In this study, two efficient subpixel accuracy improvement methods are applied to refine the MCC-based motion extraction algorithm.

### 3.2. Modified Taylor Approximation Refinement

After the motion extraction using MCC-based algorithm, we assume that the template image f(x,y) is moving towards the object image g(x,y) according to the calculated horizontal and vertical integer level displacements (Δx,Δy). Eventually, the distance between the shifted template f(x+Δx,y+Δy) and the object image g(x,y) can be regard as less than one pixel. Suppose that $f\left(x+\Delta x,y+\Delta y\right)=f_{s}\left(x,y\right)$, the relationship between $f_{s}\left(x,y\right)$ and g(x,y) can be expressed as

(7) $$g\left(x,y\right)=f_{s}\left(x+\delta x,y+\delta y\right)$$

Given that both δx and δy are assumed to be less than one pixel, Equation (7) can be approximated using the first order Taylor series approximation:

(8) $$g\left(x,y\right)=f_{s}\left(x+\delta x,y+\delta y\right)\approx f_{s}\left(x,y\right)+\delta x\frac{\partial }{\partial x}f_{s}\left(x,y\right)+\delta y\frac{\partial }{\partial y}f_{s}\left(x,y\right)$$

with two unknowns, δx and δy in one equation, the linear least-squares (LS) estimator minimizes the square error as

(9) $$\min_{\delta x,\delta y}\Phi \left(\delta x,\delta y\right)=\min_{\delta x,\delta y}\sum_{x,y}{\left[g\left(x,y\right)-f_{s}\left(x,y\right)-\delta x\frac{\partial }{\partial x}f_{s}\left(x,y\right)-\delta y\frac{\partial }{\partial y}f_{s}\left(x,y\right)\right]}^{2}$$

The minimum of Φ(δx,δy) can be found from its critical points, where its derivatives with respect to δx and δy are both zero:

(10) $$\begin{array}{c} \frac{\partial \Phi (\delta x,\delta y)}{\partial \delta x}=\sum_{x,y}\left[\delta x{\left(\frac{\partial f_{s}}{\partial x}\right)}^{2}+\delta y\left(\frac{\partial f_{s}}{\partial x}\frac{\partial f_{s}}{\partial y}\right)+\left(f_{s}-g\right)\frac{\partial f_{s}}{\partial x}\right]=0\\ \frac{\partial \Phi (\delta x,\delta y)}{\partial \delta y}=\sum_{x,y}\left[\delta y{\left(\frac{\partial f_{s}}{\partial y}\right)}^{2}+\delta x\left(\frac{\partial f_{s}}{\partial x}\frac{\partial f_{s}}{\partial y}\right)+\left(f_{s}-g\right)\frac{\partial f_{s}}{\partial y}\right]=0 \end{array}$$

Equation (10) can be rewritten in matrix form:

(11) $$\left[\begin{array}{cc} \sum_{x,y}{\left(\frac{\partial f_{s}}{\partial x}\right)}^{2} & \sum_{x,y}\frac{\partial f_{s}}{\partial x}\frac{\partial f_{s}}{\partial y}\\ \sum_{x,y}\frac{\partial f_{s}}{\partial x}\frac{\partial f_{s}}{\partial x} & \sum_{x,y}{\left(\frac{\partial f_{s}}{\partial y}\right)}^{2} \end{array}\right]\left(\begin{array}{c} \delta x\\ \delta y \end{array}\right)=\left[\begin{array}{c} \sum_{x,y}\left(g-f_{s}\right)\frac{\partial f_{s}}{\partial x}\\ \sum_{x,y}\left(g-f_{s}\right)\frac{\partial f_{s}}{\partial y} \end{array}\right]$$

In practice, the image derivatives can be approximated using numerical differentiation. Here, the finite difference is applied for the computation of partial derivatives. By solving Equation (11), the refined subpixel level displacement can be eventually written as

(12) x=Δx+δx,y=Δy+δy

At this point, the integer level motion extraction obtained by MCC-based algorithm can be effectively improved to subpixel level through the above steps.

It is noticed that all the derivations above are based on the assumption that both δx and δy are less than one pixel, which makes the Taylor approximation proper and valid. However, the actual distance between the shifted template $f_{s}\left(x,y\right)$ and the object image g(x,y) will not always meet the requirement of this assumption. In the case that the calculated displacement between $f_{s}\left(x,y\right)$ and g(x,y) is more than one pixel in the horizontal or vertical direction, one-step Taylor approximation may result in inaccurate results and lead to small errors in the refined motion curves. Although this phenomenon could be alleviated by increasing the sampling rate of CCD sensor in practice, it is not wise to increase computing burden for every motion extraction step in real-time measurement, especially when most calculations have already satisfied with the assumption. Therefore, a rounding-iterative operation is proposed to improve Taylor approximation in this study.

**Figure 4:**
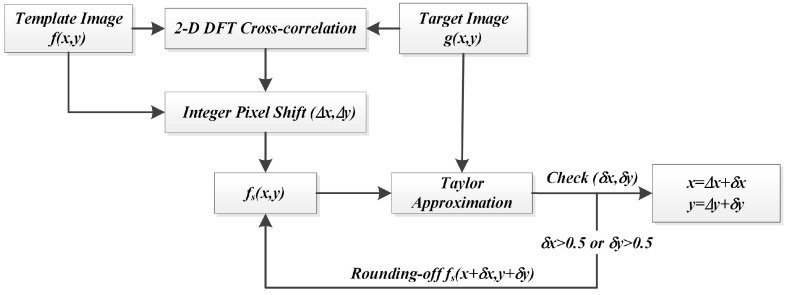
Flowchart of refinement using Taylor approximation with the reformative iteration operation.

The flowchart of the modified rounding-iterative Taylor approximation algorithm is shown in Figure 4. For each subpixel refinement step, the calculated δx and δy are set by rounding to the nearest integers until both δx and δy are less than 0.5 pixel. This modification is so simple without introducing any unnecessary computation and pixel interpolation. The modified algorithm naturally executes with a high degree of automation because the improvement brings no additional parameter that requires specification. Moreover, the rounding-off operation also avoids the subpixel interpolation computation when the template image is updated. This advantage makes the algorithm much faster than conventional iterative optical flow.

**Figure 5:**
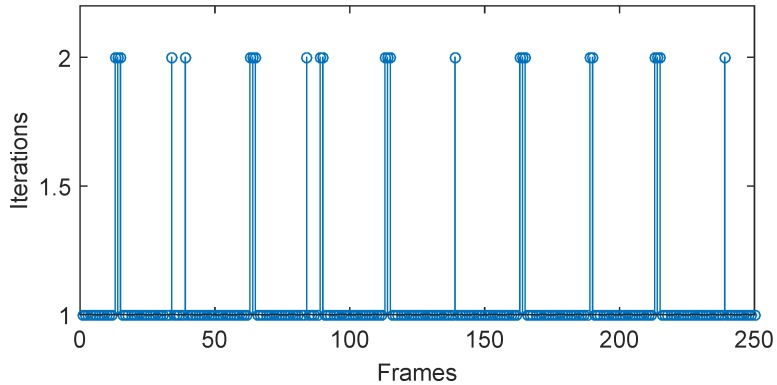
Number of iterations using the modified Taylor approximation refinement in the simulation test.

**Figure 6:**
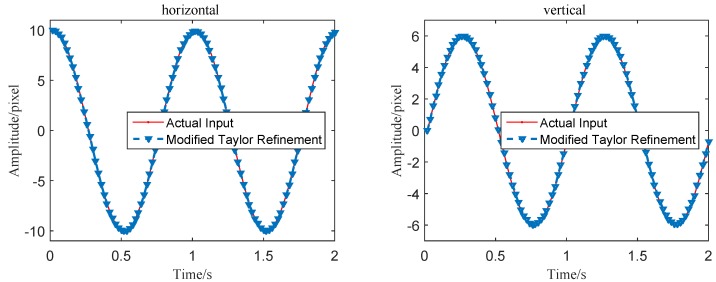
Comparisons of displacement extraction results between the actual input and the MCC with modified Taylor refinement.

Figure 5 shows the number of iterations using the modified Taylor approximation refinement in the simulation test. It can be seen that most motion extraction steps are accomplished with one rounding-iterative Taylor approximation operation, and rest calculations are completed within two rounding-iterative Taylor approximation operations. As is shown in Figure 6, the refined vibration signals in both vertical and horizontal directions are obviously smoother than the curves obtained by using integer level MCC-based algorithms. Quantitive error and time-consuming analyses will be discussed at the end of this section.

### 3.3. Subpixel Localization Refinement

During the derivation of the modified Taylor approximation refinement algorithm, the deduction is successful only when brightness constancy holds exactly. Like traditional optical flow estimation, the proposed rounding-iterative coarse-to-fine refinement method may become invalid under large illuminance vibration environments [37]. Therefore, another subpixel localization refinement approach which is insensitive to illuminance changes is introduced. Different from the modified Taylor approximation refinement, quadratic surface fitting the peak point and values around in the cross-correlation matrix is also an effective method to improve the measurement accuracy of the integer level MCC-based algorithm.

In our proposed algorithm, the zero-frequency component is firstly moved to the center of 2D DFT cross-correlation results. Eight points around the coordinates of the maximum value in the cross-correlation matrix and the maximum cross-correlation peak are used to realize the quadratic surface fitting. Although curve fitting a bigger region may obtain more accurate estimation, the complexity and quantity of computation also increase. The quadratic surface equation that was fitted by the nine points can be written as:

(13) $$S\left(x,y\right)=a_{0}+a_{1}x+a_{2}y+a_{3}x^{2}+a_{4}xy+a_{5}y^{2}$$

The coefficients a(i=0…5 can be estimated through a pseudo-inverse computation after these nine points are substituted into the quadratic surface equation:

(14) $$\left[\begin{array}{ccc} S(-1,1) & S(0,1) & S(1,1)\\ S(-1,0) & S(0,0) & S(1,0)\\ S(-1,-1) & S(0,1) & S(1,-1) \end{array}\right]=\left[\begin{array}{ccc} CC(x_{i}-1,y_{i}+1) & CC(x_{i},y_{i}+1) & CC(x_{i}+1,y_{i}+1)\\ CC(x_{i}-1,y_{i}) & CC(x_{i},y_{i}) & CC(x_{i}+1,y_{i})\\ CC(x_{i}-1,y_{i}-1) & CC(x_{i},y_{i}-1) & CC(x_{i}+1,y_{i}-1) \end{array}\right]$$

where CC is the cross-correlation matrix between 2D DFT of the template image and object image and $\left(x_{i},y_{i}\right)$ are the coordinates of the maximum value in CC . After obtaining the coefficients of the quadratic surface, the extreme point of the equation can be easily calculated by solving the following equations:

(15) $$\frac{\partial S(x,y)}{\partial x}=0,\frac{\partial S(x,y)}{\partial y}=0$$

The extreme value of the quadratic surface equation can be solved as:

(16) $$x_{s}=\frac{2a_{1}a_{5}-a_{2}a_{4}}{a_{4}^{2}-4a_{3}a_{5}},y_{s}=\frac{2a_{2}a_{3}-a_{1}a_{4}}{a_{4}^{2}-4a_{3}a_{5}}$$

**Figure 7:**
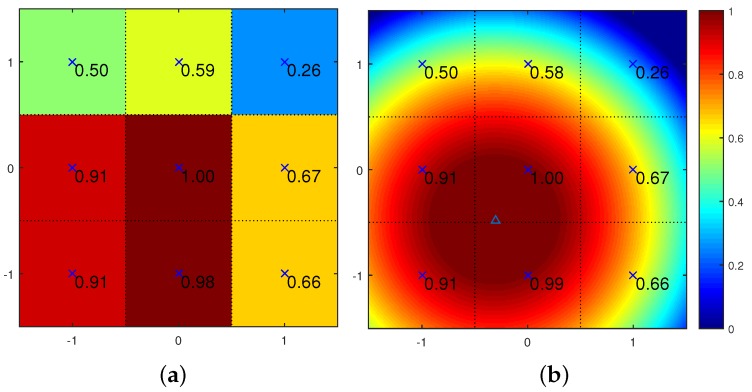
Example of the quadratic surface fitting refinement: (**a**) nine integer level cross-correlation values for curve fitting; (**b**) the fitting quandratic surface, and the triangle marker is the refined subpixel estimation of the best-matching location.

To illustrate this quadratic surface fitting vividly, a simple example between the template image (cut from the 1st frame in the simulation test) and the object image (cut from the 60th frame in the simulation test) are shown in Figure 7. Since the cross-correlation values around the maximum are quite close to each other, a normalized operation is helpful to clear observation. The original best-matching cross-correlation point and eight fitting points around are shown in Figure 7a, and the calculated quadratic surface is given in Figure 7b. The new extreme point marked with a triangle marker can be considered as a better estimation of the best-matching location.

**Figure 8:**
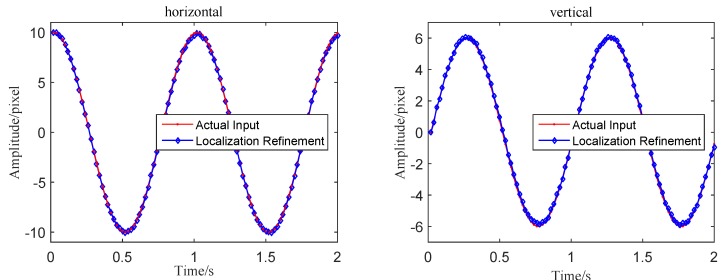
Comparisons of displacement extraction results between the actual input and the MCC with subpixel localization refinement.

Using the 2D DFT cross-correlation values, this subpixel improvement approach naturally shows strong robustness to the effect of illumination. This method can be appropriately used to refine the integer level vibration signals in the simulation. In Figure 8, the obtained wave shapes are much smoother and match well with the actual inputs compared with the MCC-based results. Therefore, the proposed surface fitting algorithm also improves the extracting accuracy successfully.

### 3.4. Quantitive Analysis

Quantitive analyses on error performance and time-consumption are given at the end of this section. In this part, another efficient subpixel image registration algorithm, namely, the upsampled cross-correlation (UCC) algorithm is also applied to the simulation test for comparison. With an improvement over the FFT upsampling approach, the UCC algorithm can achieve subpixel image registration with the same accuracy as the traditional FFT upsampling with greatly reduced computational time and memory requirements. As an advanced subpixel image registration technique, the UCC algorithm allows resolution adjustment by changing the upsampling factor. Here, the upsampling factor (usfac) for UCC algorithm are set as 10 and 100 for subpixel level of 0.1 pixel and 0.01 pixels, respectively.

Quantitive results regarding the tracking error and computation time are given in Table 1. To further evaluate the error performance, the normalized root mean squared error is once again introduced as

(17) $$NRMSE=\frac{\sqrt{\frac{1}{n}\sum_{i=1}^{n}{\left(a_{i}-b_{i}\right)}^{2}}}{b_{max}-b_{min}}\times 100\%$$

where *n* donates the frame number and *a* and *b* refer to the measured displacement data and the true movement values, respectively. The results indicate that the integer level MCC-based algorithm has the highest NRMSE of 1.52%. All of the four subpixel level motion extraction algorithms improve the signal accuracy in the simulation test successfully. With the acceleration of the upsampling factor, the motion extraction results using the UCC algorithm also get better. From the data, both of the proposed two subpixel refinement algorithms execute with better efficiency than the UCC algorithm with satisfactory error performance. The MCC-based algorithm with modified Taylor approximation refinement has the lowest NRMSE of 0.51%, and the MCC algorithm with the localization refinement costs only 2.84 ms in one extraction computation.

Because of the satisfactory performance in working efficiency and accuracy in the simulation test, both the two refined algorithms have been integrated in to the high-speed vision-based sensor system described in Section 2. The entire sensor system is implemented based on Qt and OpenCV libraries and can realize the dynamic vibration measurement of actual structures in real time.

## 4. Experimental Verification and Discussion

### 4.1. Moving Platform Experiment

A laboratory experiment on a grating ruler moving platform was carried out to evaluate the performance of the two proposed refined algorithms and the developed vision-based sensor system in the laboratory environment. Using the moire fringe technology of grating and photoelectric conversion, the incremental grating displacement sensors widely act as a high accuracy contact displacement measurement tool with numerous advantages, such as stability, reliability and high accuracy. In this experiment, motion extraction results using the developed sensor are compared with the results measured by the grating ruler displacement sensor.

**Figure 9:**
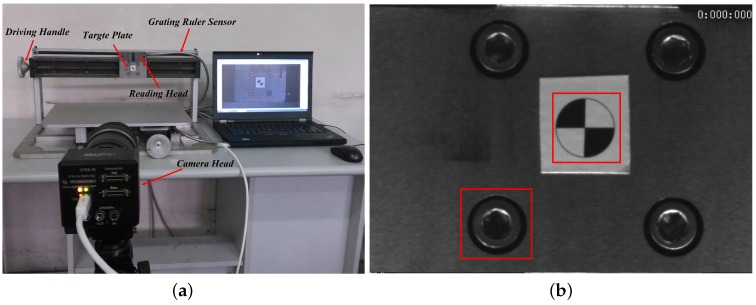
Experiment setup in moving platform experiment: (**a**) experimental device; (**b**) the selected artificial target and natural structure target.

Figure 9a shows the experiment equipment. From the figure, the grating ruler displacement sensor was installed on the moving table platform with its reading head moving synchronously with the target plate in horizontal direction. With this structure, the vibration of target plate can be recorded by the grating ruler displacement sensor and the vision-based sensor simultaneously. The camera head of the vision-based sensor is installed on a tripod for steady output. The video camera was placed 1 m away from the table along the optical axis so that the target plate can always exist in the imaging plane. The sampling frequency of the grating ruler sensor was set to be 20 Hz, and the grating pitch was 0.02 mm with a resolution of 1 μm. The camera captured the moving target at a resolution 400 pixel × 300 pixel with 200 fps during the shooting process. An artificial circle target with a diameter of 20 mm was pre-installed on the target plate for camera calibration.

In order to measure the life-size vibration amplitude, the actual pixel size of the pre-installed target was calculated. The result showed that 20 mm in real life corresponds to 67.4 pixels in the captured images, which means the pixel resolution would be 0.2967 mm/pixel. The guide screw was driven by a 10 s manual arbitrary input. Two images cut from the artificial circle and the natural structure feature (the screw) were selected as the templates as shown in Figure 9b. The size of both templates were 80 pixel × 80 pixel.

**Figure 10:**
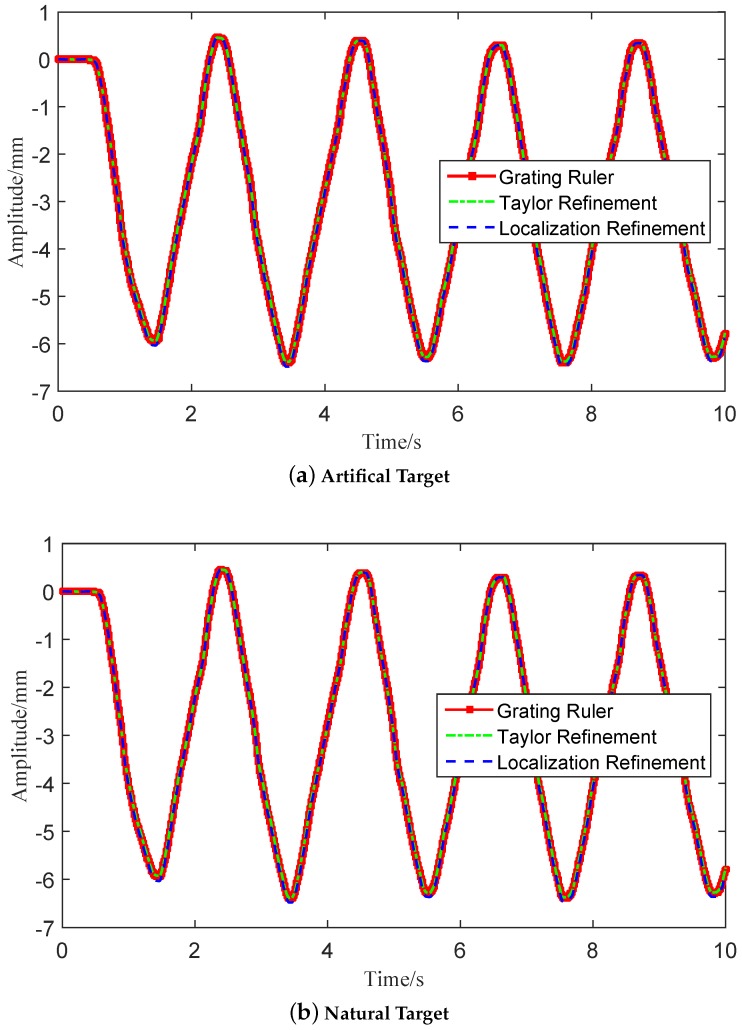
Measurement comparisons between the grating ruler sensor and the vision-based sensor: (**a**) results using an artificial target; (**b**) results using a natural structure feature.

Measurement comparisons between the grating ruler sensor and the developed vision-based sensor with refined algorithms are given in Figure 10. It can be seen that the vibration signals extracted using the modified algorithm match well with the data collected with the grating ruler sensor in both artificial target conditions and natural target conditions. Quantitive analysis about the tracking error and computation time are given in Table 2. Assuming that the grating ruler data was the true motion signal, the modified Taylor refinement algorithm achieved the lowest NRMSE of 0.61% and NRMSE of the localization refinement algorithm was 0.73%. The localization refinement algorithm spent only 0.94 ms in one motion extraction step, which is proven to be more efficient than the Taylor refinement algorithm (1.21 ms). Furthermore, both of the two modified algorithms are at least five times faster than the traditional UCC algorithm and execute with better error performance.

### 4.2. Vibration Analysis of Sound Barrier

The sound barrier, namely the sound wall or noise barrier, is a platy structure that is designed to protect inhabitants on both sides of a railway from noise pollution. However, when a high-speed train is passing by, this platy structure may suffer from strong suction caused by the train. In practice, the sound barriers along the high-speed railway often fall on the train railway track because of the fatigue of materials or assembly problems caused by the aroused vibration. To improve the design of the sound barrier and increase its working life, the vibration status should be measured the first time the high-speed train is passing by. However, installing the traditional measurement devices or attaching a pre-designed target panel to the barriers that are installed on the suspension viaduct is difficult. The vision-based vibration analysis system is a suitable alternative to accomplish this non-contact measurement.

**Figure 11:**
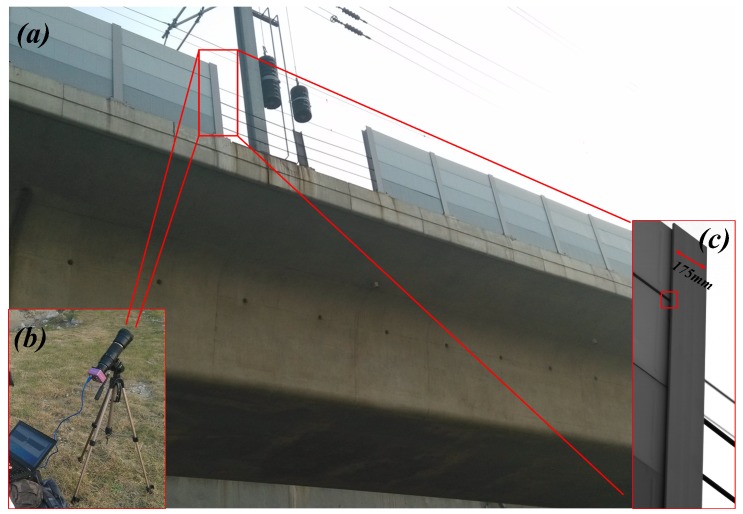
Sound barrier experiment setup: (**a**) the experiment environment; (**b**) experimental setup; (**c**) Image captured by the developed visual measuring system.

To validate the effectiveness of the proposed algorithms in extracting the vibration signal of actual large-scale structures, a field test was carried out to analyze the vibration of the sound barriers in KunShan City, which is an important railway hub city in East China. As is shown in Figure 11, the high-speed camera system was placed on the ground below the viaduct and the distance between the camera head and the barrier target was about 30 m. The vision-based sensor recorded video containing the vibration signal of the sound barrier at a sampling frequency of 232 fps when a high-speed train was passing by.

The image captured by the camera is displayed in Figure 11c. Seams and edges in the captured video can be selected as ideal tracking templates for vibration extraction. The position of the template, of which the size is 60 pixels × 60 pixels, is shown as the red box in Figure 11c. The sound barriers are supported by I-beams in which actual sectional dimensions are 175 mm × 175 mm. In the captured video, 175 mm in real life corresponds to about 225 pixels in the captured images, which means that the pixel resolution can be calculated as 0.78 mm/pixel. During the analysis process, the influence caused by the deformation of the barrier can be ignored since the deformation is very small compared to the vibration. The distance between the camera head and the bridge pier is about 28 m. The height of the railway bridge is about 12 m and the height of sound barrier is 2.15 m. The tilt angle can be calculated at about 26°, which means the error rate caused by the tilt angle is about 0.6%∼0.8% [6]. Because the amplitudes are relatively small and the errors have little influence on frequency information [6,25], the errors are considered acceptable.

**Figure 12:**
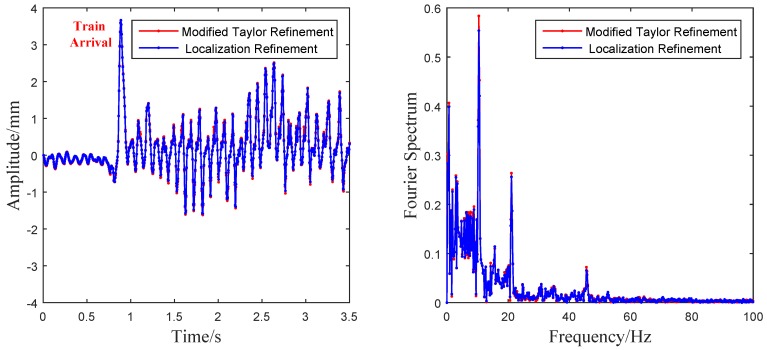
Vibration extraction results and their Fourier spectrums in the sound barrier experiment.

The vibration amplitude and its Fourier spectrum in the horizontal direction is shown in Figure 12. Both of the two modified algorithms clearly obtained similar results in terms of the time-domain wave shapes and frequency-domain spectrums. The vibration time history clearly displayed the wave shapes when the train passed by, and the moment of train arrival is marked red in Figure 12. Three obvious spectral peaks, 10.42 Hz, 21.07 Hz and 45.77 Hz, can be observed in the Fourier spectrums. In reality, it is difficult to extract the natural frequencies of a bridge from its train-induced dynamic responses and the bridge response is dominated by the excitation frequency associated with the train passing [38,39]. Because the natural frequencies of the sound barrier are far away from the excitation, frequency from the train (less than 5 Hz) and the frequency of wind load caused by the train carriages is about 2 Hz ∼ 4 Hz. The frequencies 10.42 Hz, 21.07 Hz and 45.77 Hz are considered excited by the pulsed wind excitation caused by the locomotive [40]. Thus, these peaks can be considered as the characteristic frequencies of the measured sound barrier. Moreover, it is worth mentioning that the two modified algorithms run very fast in the field test. The elapsed time of each extraction using both of the two algorithms was less than 1.5 ms. So far, the dynamic characteristic of the sound barrier, which was previously difficult to detect before, can be easily analyzed with the vision-based sensor system designed in this paper.

### 4.3. Discussion

From the analysis of the simulation and experiments above, we can see that both of the two subpixel refinement approaches successfully improve measurement accuracy of the traditional MCC-based algorithm. Different from the UCC algorithm, which improves the FFT upsampling approach, the modified Taylor and the localization refinement algorithms provide more efficient alternatives in vision-based vibration measurements. Among these two proposed algorithms, the algorithm refined by rounding-iterative Taylor approximation can get more accurate results than the algorithm refined by subpixel localization and the algorithm refined by subpixel localization is more efficient than the algorithm refined by rounding-iterative Taylor approximation.

Unlike phase-based optical flow [27,28,29,30], which can extract motion signals of all points by using changes of local phase, these refined subpixel image registration algorithms extract motion signals through the use of the cross-correlation relationship between template image and object image. Therefore, these image registration algorithms can only extract motion signals of a certain area on the target, and mode shapes of structures cannot be detected directly from the captured video. However, without calculating velocity information, the proposed image registration algorithms extract pixel displacement information directly. Because these algorithms do not need to specify coefficients for spatial band-pass filter and have no additional parameter that requires specification, the proposed methods naturally execute with a high degree of automation and are much faster than phase-based optical flow. These characteristics make the proposed methods more appropriate choices for real-time measurement.

Based on the assumption of brightness constancy or intensity conservation, the modified Taylor refinement algorithm works well on the condition that there are no specularities or secondary illumination (shadows or inter-surface reflection). However, similar to the traditional optical flow, large illumination vibrations may still have influence on the measurement results and lead to errors. Fortunately, the proposed Taylor refinement algorithm performs well in both the laboratory environment and in the wild tests. It is noticed that, the same as most cross-correlation based algorithms, the subpixel localization refinement MCC-based algorithm is naturally insensitive to illumination changes when satisfactory image quality can be guaranteed. This characteristic makes this algorithm a better choice in large illumination vibration conditions. However, poor conditions such as illumination fluctuation, partial template occlusion and background disturbance may seriously influence image quality and make this cross-correlation based algorithm invalid [41].

Different from the time-domain motion extraction algorithm, such as the normalized cross-correlation (NCC), which locates the integer level vibration results by calculating the cross-correlation values for many times, the algorithm in this paper searches the integer level results by locating the maximum value in the cross-correlation matrix between the 2D DFT of the template image and object image, which means the rough search can be accomplished in one calculation. This advantage makes the refined algorithms much faster than the conventional time-domain methods. It is worth mentioning that use of these algorithms gives accurate vibration signals when the size of the selected template is large enough relative to the motion amplitude [32]. In practical application, a relatively large template will be helpful in obtaining better extraction results.

The high-speed vision sensor system in this study is supported with a camera tripod. However, the environmental disturbances such as wind disturbances may still lead to camera vibrations and bring unwanted errors. The developed sensor system can only meet the real-time measurement under a frequency sampling below 500 Hz, limited by the CCD device we can access. Future works will be focused on improving the robustness of the modified Taylor refinement algorithm under large illumination changes, reducing the environment error and developing sensor systems for high frequency sampling over 500 Hz.

## 5. Conclusions

This study describes two efficient modified motion extraction algorithms to measure structure vibration using a high-speed digital camera. The normalized root-mean-square errors of two images are introduced in this paper. The integer level vibration signal can be obtained by calculating the cross-correlation matrix between the 2D DFT of template image and the object image and locating the maximum value in the cross-correlation matrix. Two proposed approaches, the modified Taylor approximation and the subpixel localization, are used to refine the integer level motion extraction results on the subpixel level. From the simulation tests and experiments in laboratory conditions and the real environment, we can see that both of these two modified algorithms can extract vibration signals with impressive efficiency and satisfactory error performance. The elapsed time of each extraction using both of the two algorithms is obviously less than the compared algorithm, namely the UCC algorithm. This advantage makes the vision-based sensor system realize real-time video processing. The motion platform experiment demonstrated the accuracy of the dynamic displacement measurement by comparing the results of the high-speed camera system with those of a conventional sensor of a grating ruler. The vibration analysis of the sound barrier further displayed the reliability of the proposed vision-based sensor system in long-distance real-life structure measurements.

## Figures and Tables

**Table 1:** Error and time consumption comparisons in the simulation test.

Motion Extraction Algorithm	NRMSE (%)	Time_*avg*_ (ms)	Time_*total*_ (s)
MCC	1.52	2.77	0.69
UCC (*usfac* = 10)	0.61	16.12	4.03
UCC (*usfac* = 100)	0.57	19.39	4.85
MCC with Modified Taylor Refinement	0.51	3.49	0.87
MCC with Localization Refinement	0.57	2.84	0.71

**Table 2:** Error and time consumption comparisons in the moving platform experiment.

Motion Extraction Algorithm	NRMSE (%)	Time_*avg*_ (ms)	Time_*total*_ (s)
UCC (*usfac* = 100)	0.75	6.38	12.76
MCC with Modified Taylor Refinement	0.61	1.21	2.42
MCC with Localization Refinement	0.73	0.94	1.88
